# Supplementary material for: No-reference panoramic image quality assessment based on multi-region adjacent pixels correlation
Source: PLoS One. 2022 Mar 28;17(3):e0266021. doi: 10.1371/journal.pone.0266021 (PMC8959172; doi:10.1371/journal.pone.0266021)
Supplement: Attachment — Submitted filename: PONE-D-21-39405.pdf [file pone.0266021.s001.pdf]

PONE-D-21-39405

The author proposed an novel panoramic image quality assessment based on multi-region adjacent pixels correlation to address the problem that the quality of panoramic image cannot be fully reflected due to the local division of panoramic image. The motivation is sufficient, and the method is described in detail. At the same time, a series of ablation and comparative experiments are conducted to verfiy the effectiveness of this method. Finally, please refer to the following revision for modification and explanation.

Minor Revision:

1. The author should clarify the different between the panoramic image and the normal image. Specifically, the reviewer suggests that the Introduction (line 2-7) can be rewritten in detail.

2. The font of I_{i+1,j} in Fig 1 is different from other font in this manuscript.

3. The author should clarify the parameter of the SVR such as "Penalty coefficient".

4. The size of Fig 7 should be enlarged.
