# Supplementary material for: No-reference panoramic image quality assessment based on multi-region adjacent pixels correlation
Source: PLoS One. 2022 Mar 28;17(3):e0266021. doi: 10.1371/journal.pone.0266021 (PMC8959172; doi:10.1371/journal.pone.0266021)
Supplement: Attachment — Submitted filename: response_to_comments_of_PONE-D-21-39405.docx [file pone.0266021.s002.docx]

**Response to Reviewers’ Comments (****PONE-D-21-39405)**

We thank the editor and the reviewers for their insightful comments, and we have revised the manuscript based on their suggestions. Those comments are all valuable and very helpful for revising and improving our manuscript, as well as providing important guiding significance to our research. We have studied the comments carefully and have made the corrections, and we hope meet with approval. Detailed replies to their comments and how we have taken care of these points in the revised manuscript are given below.

**The responses to the Editor’s comments are listed in the following.**

**General comment from the editor:**

Thank you for submitting your manuscript to PLOS ONE. After careful consideration, we feel that it has merit but does not fully meet PLOS ONE’s publication criteria as it currently stands. Therefore, we invite you to submit a revised version of the manuscript that addresses the points raised during the review process.

**Response**:

We appreciate the preciseness of the editor. According to the editor and the reviewers’ comments, we have tried our best to revise our manuscript carefully. Note that we have removed the funding information from the Acknowledgement section. In the following, all reviewers’ comments and our responses are in black, and all changes that have been made to the manuscript are in blue.

**The responses to Reviewer #1’s comments are as follows.**

**General comment:**

In this paper, a novel statistical feature is proposed for panoramic image quality assessment, and its validity is fully demonstrated, and the method is introduced clearly. Generally, this work is interesting and reasonable. However, this paper can be improved from the following aspects.

**Response:**

We appreciate the approval of the reviewer. According to the comments of the reviewer, we have added some details in the revised manuscript. The suggestions significantly help us to improve our manuscript.

**Detail Comments:**

**Comment 1:**

In Section 3, the proposed method is presented in four parts, for each of them please use mathematical formulas to summarize the processing.

**Response 1:**

We are grateful to the reviewer for the good suggestion. Although we presented the corresponding equations in the text of each part, it is still not intuitive enough for readers. To highlight and summarize each part, according to the suggestion of the reviewer, we have added the corresponding mathematical formulas in Fig 5, i.e., the framework diagram.

**Fig 5.** Framework of quality assessment method based on multi-region adjacent pixel correlation feature.

Besides, we have also adjusted the contents about residual truncation (i.e., Eq. 1).

“It should be noted that since most of the residual values are concentrated in a small range, we set a threshold *T*=2 for difference map $\mathbf{R}$ to reduce the residual range:

$$\begin{aligned} \left\{ \begin{aligned} R_{i,j}=-T,R_{i,j}<-T \\ R_{i,j}=T,R_{i,j}>T \end{aligned} \right.\#\left( 1 \right) \end{aligned}$$

” (Please see the last paragraph in Section 3.1)

**Comment 2:**

In Eq.5, the authors propose a nonlinear mapping of the predicted scores; please explain the reasons for this approach here.

**Response 2:**

We appreciate the preciseness of the reviewer. The nonlinear mapping we used is a common and typical fitting model, which is recommended by Video Quality Experts Group [R1]. According to the comment of the reviewer, we have revised some sentences to explain the reasons and added a new citation [R1] ([19] in the revised manuscript) in the reference list.

“Note that the proposed algorithm follows the recommendations given in [19] and uses a 5-parameter logistic function to fit the predicted results, as shown in Eq. (5).” (Please see the second paragraph in Section 4.1)

19. Antkowiak J, Baina TJ. Final report from the video quality experts group on the validation of objective models of video quality assessment march. In: VQEG Meeting; 2000. (Please see the reference list)

**The response to Reviewer #2’s comments is presented in the following.**

**General comments from the reviewer:**

The author proposed an novel panoramic image quality assessment based on multi-region adjacent pixels correlation to address the problem that the quality of panoramic image cannot be fully reflected due to the local division of panoramic image. The motivation is sufficient, and the method is described in detail. At the same time, a series of ablation and comparative experiments are conducted to verify the effectiveness of this method. Finally, please refer to the following revision for modification and explanation.

**Response:**

We appreciate the reviewer for providing us the opportunity to revise the manuscript. According to the reviewers’ comments, we have tried our best to revise our manuscript carefully. In the current version of our manuscript, we have clarified the differences between panoramic images and normal images, revised the contents of some figures, and clarified some parameters of the SVR. We thank the valuable comments from the reviewer.

**Comment 1:**

The author should clarify the different between the panoramic image and the normal image. Specifically, the reviewer suggests that the Introduction (line 2-7) can be rewritten in detail.

**Response 1:**

We are grateful to the reviewer for the good suggestion. According to the suggestion of the reviewer, we have rewritten the first paragraph in Section 1, where citation [1] is newly added.

“Compared with normal planar images, panoramic images adopt the image stitching technique and can achieve free viewing in various directions, which provide users an immersive experience with the help of head-mounted displays (HMDs). In practical, the operations, including stitching, blending, projecting and encoding, inevitably lead to quality degradation of panoramic images, and further cause discomfort and physical illness [1]. Therefore, it is urgent for panoramic images to propose an accurate quality assessment algorithm in the future immersive applications.” (Please see the first paragraph in Section 1)

1. Singla A, Fremerey S, Robitza W, Raake A. Measuring and comparing QoE and simulator sickness of omnidirectional videos in different head mounted displays. In: 2017 Ninth International Conference on Quality of Multimedia Experience (QoMEX); 2017. p. 1–6. (Please see the reference list)

**Comment 2:**

The font of I_{i+1,j} in Fig 1 is different from other font in this manuscript.

**Response 2:**

We appreciate the preciseness of the reviewer. As the reviewer mentioned, the font of *I_i+1,j_* and *I_i,j_* should be in italic with Times New Roma. Therefore, we have modified Fig 1 in the revised manuscript.

**Fig 1.** The probability distribution of adjacent pixel pair (*I_i,j_*, *I_i+1,j_*).

(Please see Fig 1 in the revised manuscript)

**Comment 3:**

The author should clarify the parameter of the SVR such as "Penalty coefficient".

**Response 3:**

We appreciate the preciseness of the reviewer. As the manuscript described, we employ the SVM function “fitrsvm” of MATLAB 2018a to implement the SVR model. Note that the penalty coefficient and the gamma as the hyper-parameters are default in our experiments. Therefore, we have added some sentences in the revised manuscript to clarify this point.

“In the experiment, the SVM function “fitrsvm” of MATLAB 2018a is selected for training, where the radial basis function (RBF) is selected as the kernel function and other parameters keep the default configurations.” (Please see the last paragraph in Section 4.1)

**Comment 4:**

The size of Fig 7 should be enlarged.

**Response 4:**

We are grateful to the reviewer’s comment. Since the maximum size of figure is restricted, we have enlarged all sub-figures in Fig 7.

**Fig 7.** Scatter diagrams for the pairs of MOS score and objective score of different algorithms.

(Please see Fig 7 in the revised manuscript)
